# The Effect of Reactive Ionic Liquid or Plasticizer Incorporation on the Physicochemical and Transport Properties of Cellulose Acetate Propionate-Based Membranes

**DOI:** 10.3390/polym10010086

**Published:** 2018-01-17

**Authors:** Edyta Rynkowska, Kateryna Fatyeyeva, Joanna Kujawa, Krzysztof Dzieszkowski, Andrzej Wolan, Wojciech Kujawski

**Affiliations:** 1 Faculty of Chemistry, Nicolaus Copernicus University in Toruń, 7, Gagarina Street, 87-100 Torun, Poland; edyta.rynkowska@wp.pl (E.R.); joanna.kujawa@umk.pl (J.K.); dzieszko@doktorant.umk.pl (K.D.); wolan@chem.umk.pl (A.W.); 2 Normandie University, UNIROUEN, INSA Rouen, CNRS, PBS, 76000 Rouen, France; 3 Synthex Technologies Sp. z o.o., 7 Gagarina Street, 87-100 Toruń, Poland

**Keywords:** cellulose acetate propionate, reactive ionic liquid, plasticization, hydrophilic pervaporation, propan-2-ol dehydration

## Abstract

Pervaporation is a membrane-separation technique which uses polymeric and/or ceramic membranes. In the case of pervaporation processes applied to dehydration, the membrane should transport water molecules preferentially. Reactive ionic liquid (RIL) (3-(1,3-diethoxy-1,3-dioxopropan-2-yl)-1-methyl-1*H*-imidazol-3-ium) was used to prepare novel dense cellulose acetate propionate (CAP) based membranes, applying the phase-inversion method. The designed polymer-ionic liquid system contained ionic liquid partially linked to the polymeric structure via the transesterification reaction. The various physicochemical, mechanical, equilibrium and transport properties of CAP-RIL membranes were determined and compared with the properties of CAP membranes modified with plasticizers, i.e., tributyl citrate (TBC) and acetyl tributyl citrate (ATBC). Thermogravimetric analysis (TGA) testified that CAP-RIL membranes as well as CAP membranes modified with TBC and ATBC are thermally stable up to at least 120 °C. Tensile tests of the membranes revealed improved mechanical properties reflected by reduced brittleness and increased elongation at break achieved for CAP-RIL membranes in contrast to pristine CAP membranes. RIL plasticizes the CAP matrix, and CAP-RIL membranes possess preferable mechanical properties in comparison to membranes with other plasticizers investigated. The incorporation of RIL into CAP membranes tuned the surface properties of the membranes, enhancing their hydrophilic character. Moreover, the addition of RIL into CAP resulted in an excellent improvement of the separation factor, in comparison to pristine CAP membranes, in pervaporation dehydration of propan-2-ol. The separation factor β increased from ca. 10 for pristine CAP membrane to ca. 380 for CAP-16.7-RIL membranes contacting an azeotropic composition of water-propan-2-ol mixture (i.e., 12 wt % water).

## 1. Introduction

Cellulose-based polymers are commonly utilized as sustainable and green materials because of their eco-friendly nature in the production of food packaging, coating and wrapping [1,2,3,4,5]. However, the application of cellulose is restricted, due to its limited solubility related to the numerous strong hydrogen bonds between OH groups and a compact arrangement in the cellulose structure [2,6,7]. Consequently, cellulose monoesters, e.g., cellulose acetate (CA) [8,9,10,11], as well as multiesters, like, cellulose acetate propionate (CAP) [12,13,14] and cellulose acetate butyrate (CAB) [12,15], are often utilized. Compared to the cellulose monoesters, CAP and CAB show higher solubility, better stability in terms of structure, and better resistance to light [12,16]. The properties of cellulose esters depend on the number of acyl groups and their chain length, as well as on the degree of polymerization, which explains the enhanced properties of cellulose triesters compared to cellulose monoesters. However, such polymers possess drawbacks related to weak mechanical properties and poor thermal processability [17]. The use of plasticizers alters cellulose ester polymers by diminishing intermolecular forces between polymer chains, thus enhancing flexibility [15] and provoking polymer material softening due to reduced glass transition temperature (*T*_g_) and elastic modulus [17]. A good plasticizer should be characterized by a high compatibility with polymer, non-toxicity, low sensitivity to ultraviolet (UV) radiation, as well as resistance to migration and hence superior a boiling temperature and minor volatility. The effectiveness of plasticization refers to the amount of plasticizer necessary to obtain the required mechanical performance of the elaborated membrane materials. Among the most commonly and widely used plasticizers are phthalate esters, such as di-isononyl phthalate (DINP), di-isodecyl phthalate (DIDP), or dioctyl phthalate (DOP). However, their usage has been questioned because of low molecular weight and thus the possibility of migration from the polymer matrix. The released plasticizer can contaminate the air, food and drinking water, which exposes a human to poisoning. These concerns have led to a growing interest in replacing harmful phthalates by non-toxic substituents like adipic or citric acids, alkyl esters, or epoxidized triglyceride vegetable oils [15] as well as by using ionic liquids characterized by negligible volatility, slight viscosity and chemical stability [18]. Ionic liquids have been successfully employed to reveal a plasticization effect on materials. Ning et al. [18] used 1-allyl-3-methylimidazolium chloride ([AMIM][Cl]) as a plasticizer for cornstarch, simultaneously improving its conductive properties, whereas Liu et al. [19] utilized [AMIM][Cl] to obtain cellulose nanocrystals (CNCs) with tuned pliability and coloration. Matsumoto et al. [20] reported the plasticization effect on epoxy resins by addition of [21] [TFSI] ionic liquid of up to 40 wt % reflected by the decrease of Young’s modulus (650 MPa). Schmidt et al. [22] used pyrrolidinium (1-butyl-1-methyl-pyrrolidinium/BMPyr) and imidazolium (1-butyl-3-methyl-imidazolium/BMI, 1-hexyl-3-methyl-imidazolium/HMI) based ionic liquids containing hydrophobic (tris(pentafluoroethyl)trifluorophosphate/FAP, bis(trifluoromethylsulfonyl) imide/BTSI, hexafluorophosphate/PF_6_) and more hydrophilic (tetrafluoroborate/BF_4_) anions for Nafion 117 membrane modification. The incorporation of ionic liquids containing cations with flexible butyl and hexyl side chains to the Nafion matrix caused the decrease of the Young’s modulus. Rahman et al. [23] introduced ammonium-, imidazolium- and phosphonium-based ionic liquids as promising plasticizers for poly(vinyl chloride) (PVC), showing better performance than traditional phthalate plasticizers. Scrutiny of PVC doped with ionic liquids revealed that trihexyl(tetradecyl) phosphonium bis(trifluoromethane) sulfonylimide [thtdPh][Tf_2_N] ionic liquid possesses the highest leaching resistance due to its hydrophobic character, whereas trihexyl(tetradecyl) phosphonium chloride [thtdPh][Cl] ionic liquid revealed the occurrence of no migration associated with the presence of a coordinating chloride ion (Cl^−^) which successfully immobilized the ionic liquid in the polymer network [23]. 

The use of ionic liquids is a very promising and effective approach to obtaining polymeric materials with ameliorated mechanical properties, simultaneously possessing tuned physicochemical, selective and transport properties [24]. Such characteristics are crucial for the membrane materials being potentially applied in membrane-separation processes. The main objective of this work was focused on the elaboration of a phase-inversion method for the preparation of dense cellulose acetate propionate-based membranes with reduced brittleness and increased elasticity by doping the polymer with (3-(1,3-diethoxy-1,3-dioxopropan-2-yl)-1-methyl-1*H*-imidazol-3-ium bromide). The resultant CAP-RIL membranes were subsequently characterized including nuclear magnetic resonance spectroscopy (NMR), thermogravimetric analysis (TGA), and tensile tests. Evaluation of the effectiveness of the modification of the CAP with reactive ionic liquid and commercial plasticizers in terms of mechanical performances was also reported. Our interest was also focused on the impact of reactive ionic liquid and plasticizer content on the swelling properties and hydrophilic characteristics of the membrane surface. Moreover, the transport and separation properties of cellulose acetate propionate membranes with immobilized reactive ionic liquid (RIL) were assessed in the dehydration of 2-propan-ol applying pervaporation.

## 2. Materials and Methods 

### 2.1. Studied Materials

**Figure 1:**
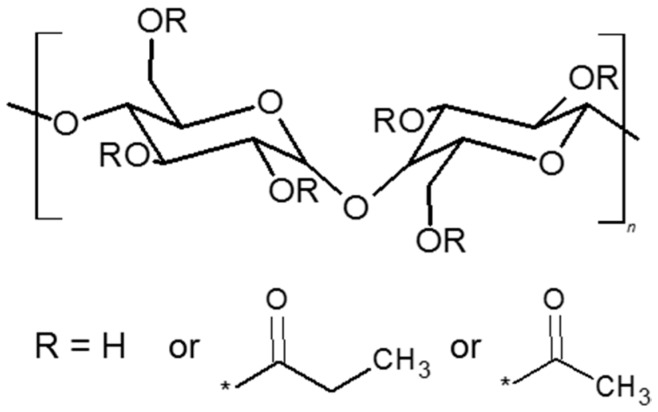
Chemical structure of cellulose acetate propionate.

**Figure 2:**
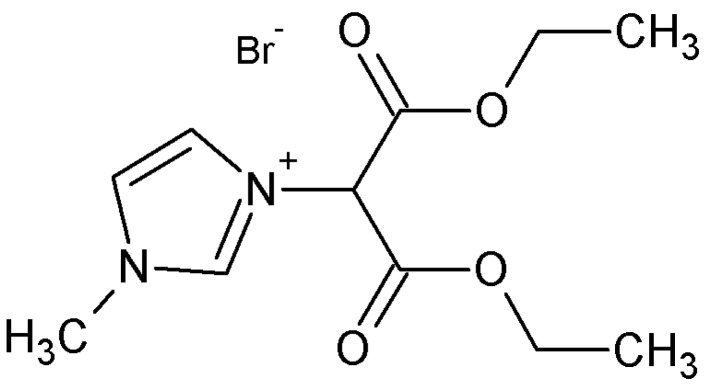
Chemical structure of reactive ionic liquid (RIL) used in this research, 3-(1,3-diethoxy-1,3-dioxopropan-2-yl)-1-methyl-1*H*-imidazol-3-ium bromide.

Cellulose acetate propionate (CAP-482-20, MW = 25,000–247,000 g·mol^−1^) was purchased from Eastman (Kingsport, TN, USA) and its chemical structure is presented in Figure 1. The reactive ionic liquid 1,3-diethoxy-1,3-dioxopropan-2-yl)-1-methyl-1*H*-imidazol-3-ium bromide (Figure 2) was synthesized using *N*-methylimidazole (Sigma-Aldrich, Poznań, Poland) and diethyl 2-bromomalonate (Sigma-Aldrich, Poznań, Poland). Solvents with the analytical reagent grade (diethyl ether, chloroform, propan-2-ol, and ethanol purchased from Avantor Performance Materials Poland S.A., Gliwice, Poland) and ultrapure water deionized by Milli-Q (18.2 MΩ·cm^−1^, Millipore^®^_,_ Fontenay-sous-Bois, France) were used. Table 1 collates chosen physicochemical characteristics of the solvents used for swelling and pervaporation measurements.

### 2.2. Synthesis of Reactive Ionic Liquid (RIL)

The RIL based on the imidazolium cation (3-(1,3-diethoxy-1,3-dioxopropan-2-yl)-1-methyl-1*H*-imidazol-3-ium) (Figure 2) was synthesized in Synthex Technologies Ltd. (Toruń, Poland) and then studied in this work. A 50 mL flask was charged with *N*-methylimidazole (0.80 mL; 10 mmol) and chloroform (10 mL). Next, diethyl 2-bromomalonate (1.70 mL; 10 mmol) was added under an argon atmosphere and the resultant yellowish solution was heated under reflux for 24 h. Subsequently, solvent was removed using a rotary evaporator and the remaining yellow liquid was washed with diethyl ether (3 × 20 mL). Residual solvents were removed under vacuum (66.7 Pa; 5 h). The synthesis-reaction yield was equal to 98.8%. The structure obtained was confirmed by NMR analysis. 1H NMR (400 MHz, Bruker Avance III spectrometer, Bruker, Rheinstetten, Germany, CDCl3) δ ppm 10.55 (s, 1 H), 7.83 (t, *J* = 1.8 Hz, 1 H), 7.58 (t, *J* = 1.8 Hz, 1 H), 7.07 (s, 1 H), 4.34 (dqt, *J* = 15.9, 7.1, 3.7 Hz, 4 H), 4.13 (s, 3 H), 1.33 (t, *J* = 7.2 Hz, 6 H). 13C NMR (101 MHz, CDCl3) δ ppm 163.11, 138.42, 123.24, 122.99, 64.16, 62.93, 37.12, 13.90.

### 2.3. Preparation of Cellulose Acetate Propionate-Based Membranes Containing RILs and Plasticizers

**Figure 3:**
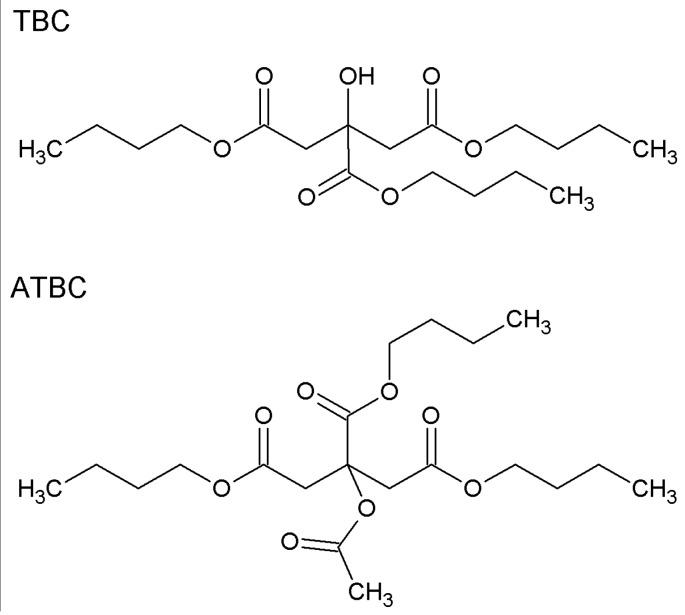
Chemical structure of tributyl citrate (TBC) and tributylacetyl citrate (ATBC).

Initially, 10 g of cellulose acetate propionate (CAP) was dissolved in 90 g of chloroform at room temperature for at least 12 h to obtain the solution used to prepare native CAP and CAP-based membranes. Next, various amounts of tributyl citrate (TBC) or tributylacetyl citrate (ATBC) (9, 23, and 50 wt %) plasticizers and RIL (9; 12.3; 16.7; 23; 28.6; 37.5; 44.4 wt %) were added to a solution of CAP in chloroform and stirred at the room temperature (21 °C ± 3 °C) for 24 h. The chemical structures of TBC and ATBC plasticizers are presented in Figure 3. In the next step, around 5 g of the solution obtained was poured on to a glass Petri dish. Subsequently, this was covered with the top half of the Petri dish and placed under the hood at an ambient temperature for a slow solvent evaporation. A phase-inversion method using the solvent evaporation technique was applied to prepare dense CAP-based membranes [26]. According to the scientific literature, the drying process can influence the membrane structure and morphology in an essential way. Albo et al. [27,28,29] investigated the impact of various drying procedures (e.g., solvent evaporation/ethanol−hexane, freeze-drying, and drying samples at room temperature/RT with an additional heating step at a higher temperature/120 °C) on the final membrane properties. It was shown that, depending on the selected procedure, it was possible to tune membrane structure. Albo et al. noticed that the use of the solvent evaporation method for implementing the ethanol−hexane system possesses the strongest impact on the membrane permeability. On the other hand, membranes dried at room temperature and subsequently at elevated temperature were the least impacted [27,28,29].

### 2.4. Characterization of Membranes

A TGA thermogravimetric analyzer (TGA Q 500, TA Instruments, New Castle, DE, USA) was used to perform TGA measurements from 25 to 800 °C under a nitrogen atmosphere (heating rate 10 °C/min and nitrogen flow rate 90 mL/min).

An Instron 5543 machine was utilized to evaluate CAP-based mechanical properties by determining the tensile deformation at a crosshead speed of 1 mm/min at 23 ± 2 °C and 43 ± 5% of relative humidity. The dimensions of the sample were the following: length 30 mm, width 5 mm, and thickness 145 ± 15 μm.

The hydrophobic/hydrophilic character of the membranes obtained was studied by the contact angle measurement realized at around 22 °C and 50% relative humidity (RH). The contact angle was determined with an accuracy of ±3° using a Multiscope apparatus (Optel, Sinzing, Germany) and applying the sessile drop method. The contact angle of each drop of water (5.1 ± 0.3 µL), glycerol (4.5 ± 0.2 µL), and diiodomethane (1.1 ± 0.1 µL) was measured after 5 s of equilibration. The surface free energy (SFE) was calculated based on the Owens–Wendt method [30]. Detailed analysis of calculated polar and dispersive components was also performed. According to the requirements of the Owens–Wendt approach, three types of testing liquids were used, i.e., polar (water), non-polar (diiodomethane), and bipolar (glycerol) [30].

### 2.5. Swelling Measurements

Gravimetrical measurements of tested membranes in contact with pure water, ethanol, and propan-2-ol at 25 °C were performed in order to evaluate the swelling behavior of membranes. Solvent uptake was estimated on the basis of the mass change between the dry membrane (W_dry_) and the membrane equilibrated in a given solvent (W_swelled_). Prior to the experiments, membrane samples were dried in the desiccator over P_2_O_5_ in a vacuum at ambient temperature for at least 48 h, after which the membranes were weighed (W_dry_). Subsequently, membrane samples were immersed into solvents, whereupon they were taken out and the excess solvent from the membranes’ surface was wiped with paper, and samples were weighed again. The latter step was repeated every 3–4 h until a constant mass of swelled membrane sample was reached (W_swelled_). The degree of swelling was calculated according to Equations (1) and (2) [31]:

(1) $${\mathrm{SD}}_{w}=\frac{W_{\mathrm{swelled}}-W_{\mathrm{dry}}}{W_{\mathrm{dry}}}\left[\frac{g\;\mathrm{solvent}}{g\;\mathrm{dry}\;\mathrm{membrane}}\right]$$

(2) $${\mathrm{SD}}_{M}=\frac{{\mathrm{SD}}_{W}}{M_{\mathrm{sol}}}\left[\frac{\mathrm{mol}\;\mathrm{solvent}}{g\;\mathrm{dry}\;\mathrm{membrane}}\right]$$

where SD_W_ and SD_M_ denote mass swelling degree and molar swelling degree, respectively; W_swelled_ and W_dry_ are the weight of the dry and equilibrated membrane, respectively; and M_sol_ is the solvent molecular mass (Table 1).

### 2.6. Pervaporation

The transport and separation properties of the CAP-RIL membranes formed were evaluated during a vacuum pervaporation (VPV) process. The experimental setup has been presented and described in detail elsewhere [31,32]. Prior to measurement, the setup was running for 1h to reach a stationary state at a given concentration and temperature. During the experiments, the membrane was in contact with feed solution containing 7–14 wt % of water in aqueous propan-2-ol mixture. All measurements were realized at 35 °C. The compositions of feed and permeate solutions were evaluated using gas chromatography (Varian 3300 gas chromatograph with a TCD detector, Varian, Inc., Walnut Creek, CA, USA) [31,32]. Borwin software (version 1.21.07, JMBS, Grenoble, France) was employed for the data acquisition and processing. The evaluation of the analytical method (limit of quantification (LOQ) and limit of detection (LOD)) is presented in our previous works [31,33]. The LOD and LOQ of water were as follows:

Water: LOD = 0.02 wt %, LOQ = 0.07 wt %

Relative standard deviations for repeatability (RSD_r_ for n = 5) and reproducibility (RSD_R_ n = 11, 4 operators) in the range of water concentrations investigated were as follows:

Water: RSD_r_ < 0.8%, RSD_R_ < 6.2%

The effectiveness of pervaporative separation was assessed by using the following parameters: total permeate flux (J_tot_)—Equation (3); mass and molar partial permeation fluxes (J_i_)—Equations (4) and (5); thickness-normalized fluxes (J_N,i_)—Equation (6); separation factor (β)—Equation (7); and thickness-normalized Pervaporation Separation Index (PSI_N_)—Equation (8) [31,34,35].

(3) $$J_{\mathrm{tot}}=\frac{{\Delta m}_{t}}{A\;\Delta t}\left[\frac{\mathrm{kg}}{m^{2}\;h}\right]$$

(4) $$J_{i}=J_{\mathrm{tot}}y_{i}\left[\frac{\mathrm{kg}}{m^{2}\;h}\right]$$

(5) $$J_{m,i}=\frac{J_{i}}{M_{i}}\left[\frac{\mathrm{mol}}{m^{2}\;h}\right]$$

(6) $$J_{N,i}=J_{i}d\left[\frac{\mu m\;\mathrm{kg}}{m^{2}\;h}\right]$$

(7) $$\beta =\frac{y_{i}/\left(1-y_{i}\right)}{x_{i}/\left(1-x_{i}\right)}$$

(8) $${\mathrm{PSI}}_{N}=J_{N,i}\left(\beta -1\right)$$

where: Δm_t_—total weight of compound in permeate (kg) collected over Δt time (h); A—area of membrane (m^2^); y, x—composition of permeate and feed; and M_i_—molar mass of i (g·mol^−1^), d—membrane thickness (μm).

## 3. Results and Discussion

### 3.1. Mechanical Properties

**Figure 4:**
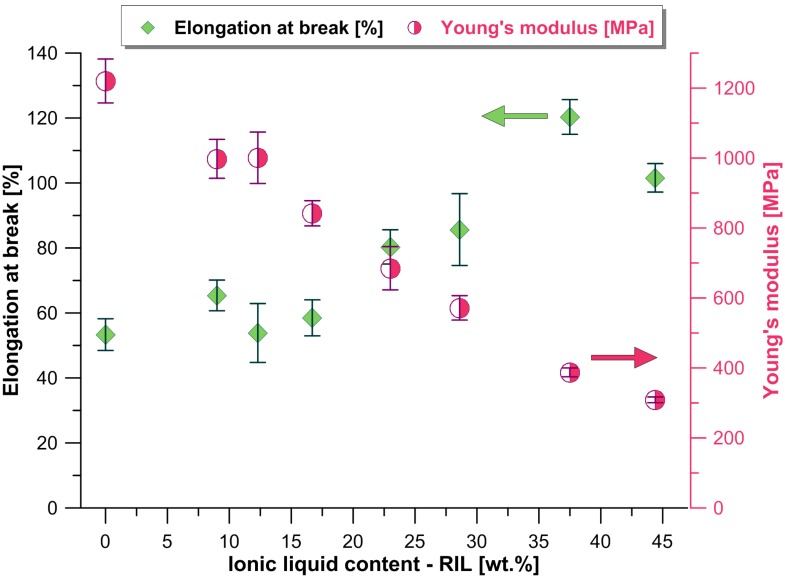
Young’s modulus and elongation at break of cellulose acetate propionate prepared with reactive ionic liquid (CAP-RIL) membranes.

The implementation of membrane separation processes in which a pressure difference is a driving force requires the application of polymeric materials with good mechanical strength and elasticity. Due to the brittleness of a native CAP membrane, the use of some kind of plasticizer is required. In order to improve the CAP membrane’s ductility, a reactive ionic liquid (RIL) as well as plasticizers (TBC, and ATBC) were used in this study. The effectiveness of the membrane’s modification was assessed based on the Young’s modulus (YM), elongation at break (ε_max_) and stress at break (σ_max_), applying standard tensile tests. The average values of YM and ε_max_ as a function of RIL content is plotted in Figure 4 for CAP-RIL membranes. Afterwards, the results obtained for CAP membranes with RIL were compared to those for membranes modified with TBC and ATBC as well as to data in the literature (Table 2).

In general, with an increasing content of RIL a rise of the elongation at break value, as well as a decrease of both stress at break and elastic modulus (YM), are observed. A remarkable improvement of mechanical properties of CAP-based membranes compared with the pristine material was observed as a result of the RIL incorporation, reflected by their reduced brittleness with simultaneous improvement of the elongation at break (Figure 4). Schmidt et al. reported that the impregnation of Nafion 117 membrane with ionic liquids based on imidazolium cation has a plasticizing effect on the polymer matrix, reflected by a decline in the Young’s modulus of impregnated Nafion membranes compared to pristine ones [22]. The improved flexibility and softness of impregnated Nafion membrane was related to the presence of ionic liquid possessing butyl and hexyl alkyl side chains of remarkable flexibility. Moreover, the incorporation of the ionic liquids weakened the hydrogen bonds between sulfonic groups [22]. The similar influence of ionic liquid incorporation on the mechanical properties of polymeric membranes was reported for sulfonated poly(ether ether ketone) (SPEEK) [36].

The mechanical properties of CAP-RIL membranes were compared with those of plasticized CAP-based membranes with TBC and ATBC plasticizers in order to assess the plasticizing efficiency of RIL with respect to commercial plasticizers (Table 2). TBC and ATBC are the environmentally friendly alternatives for commonly used plasticizers, such as phosphoric (for example, triphenyl and tricresyl phosphate) or carboxylic esters (including phthalate and citric esters) [15,37,38]. The parameters studied reflecting the mechanical properties of CAP-RIL membranes are similar to those obtained for CAP-TBC and CAP-ATBC membranes. The ductility of CAP-based membranes changed whatever compound was used (RIL or plasticizer). For example, the Young’s modulus values of membranes with content of plasticizing compound of 23 wt % were equal to 685 ± 62 MPa (CAP-23-RIL), 721 ± 22 MPa (CAP-23-ATBC), and 571 ± 20 MPa (CAP-23-TBC). On the other hand, it can be noticed that the Young’s modulus, elongation at break, and stress at break values depends on the content of the RIL used and the plasticizers. The incorporation of RIL and plasticizers (TBC, ATBC) results in the extended elongation at break of CAP-based membranes. These results are in accordance with the findings of Wojciechowska for cellulose acetate butyrate (CAB) membranes containing TBC as a plasticizer [15]. The increasing content of TBC from 25 to 35 wt % causes an increase of the elongation at break value from 24.3 ± 2.3% to 38.1 ± 4.4% [15]. At the same time, the increasing content of RIL and plasticizers (TBC, ATBC) diminishes the elastic modulus and tensile strength with respect to the pure CAP sample, which confirms that CAP-based membranes are plasticized [15].

Taking into consideration the Young’s modulus and elongation at break parameters, the incorporation of at least 9 wt % of RIL or plasticizers (TBC and ATBC) to CAP-based membrane allowed the achievement of more advantageous mechanical performance than the application of essential oils [39]. The use of 10% and 20% (*v*/*w*) of lemongrass and basil oil for CAP-based membranes did not change the elongation at break value significantly compared to the pure CAP [41] (Table 2). Moreover, it can be confirmed that the CAP-based membranes containing RIL or plasticizers (TBC and ATBC) elaborated in this work are stronger than CAP-based membranes modified with essential oils. This was confirmed by the higher stress at break values of CAP-RIL, CAP-TBC, and CAP-ATBC membranes in comparison with CAP-based membranes containing lemongrass and basil oils (Table 2). In the case of CAP-based membranes plasticized with aliphatic polyesters, such as poly(tetramethylene succinate) (PTS), poly(tetramethylene glutarate) (PTG), and dioctyl adipate (DOA) [41], the highest tested concentration of PTS equal to 27 wt % yielded lower improvement of membrane elasticity compared with CAP-9-RIL membrane, which is reflected by a more than twice the elongation achieved by CAP-9-RIL than CAP-27-PTS [41] before the sample broke. The incorporation of 9 and 23 wt % of RIL to CAP-based membranes led to the achievement of preferable mechanical properties in comparison to the addition of TBC or DOP to CAB-tetraethoxysilane (TEOS) hybrid membranes [15]. The CAB/TEOS membrane doped with 30 wt % of TBC was characterized by the best mechanical properties among the membranes tested [15] but possessed 1.5 times lower elongation at break and comparable tensile strength with CAP-23-RIL membranes elaborated in this study (Table 2). 

### 3.2. Thermal Properties

**Figure 5:**
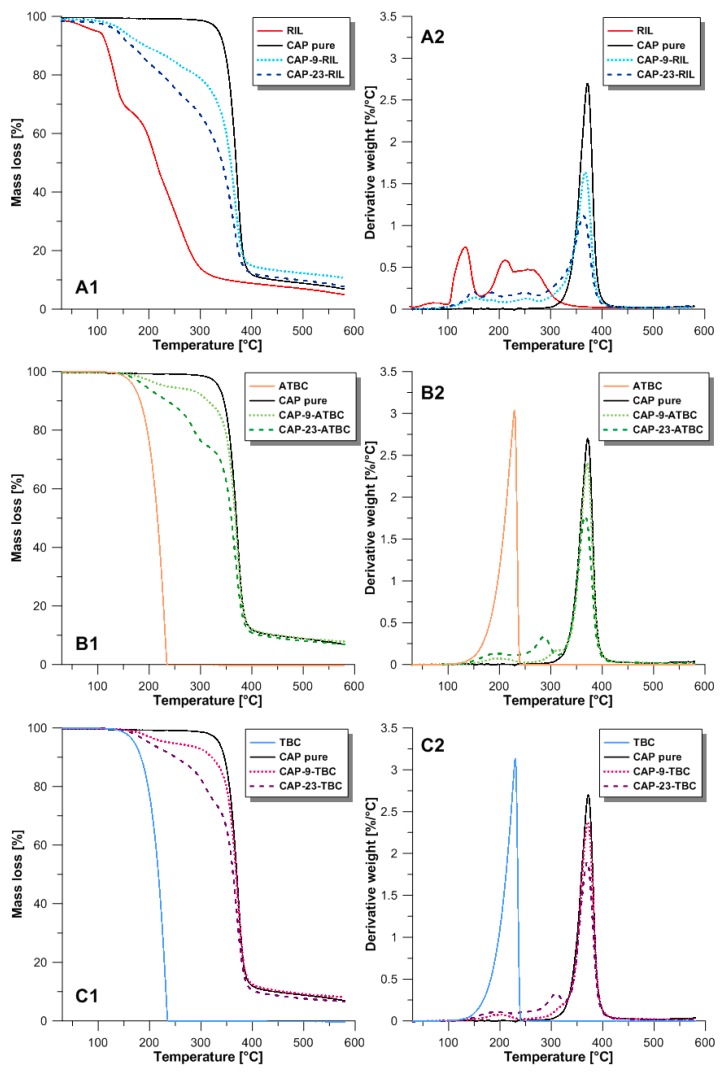
Thermogravimetric analysis (TGA) and derivative thermo-gravimetric analysis (DTG) of membranes doped by RIL (**A**); ATBC (**B**); and TBC (**C**).

The thermal properties of pure RIL, pristine CAP membrane, and CAP-based membranes modified with RIL and plasticizers (TBC and ATBC) were investigated by using thermogravimetric analysis (Figure 5). The thermal degradation profile of CAP and CAP-RIL membranes shows one degradation step for pure CAP membrane, which is related to the simultaneous degradation of acetate and propionate functional groups with a subsequent pyrolysis of the cellulose ring [39] (Figure 5A). In the case of pure RIL, four degradation steps are observed. A first peak for RIL at the onset temperature equal to 53.6 °C is present due to the release of about 5% of moisture absorbed by pure ionic liquid [42]. The subsequent steps (*T*_onset_ equal to 114.7, 192.2, 250.8 °C) are related to the decomposition of ester groups and the imidazole ring [43]. The second stage may be related to the formation of bromoethane due to the nucleophilic attack of ester groups in RIL by the bromide anion [44] (Figure 5A). Ohtani et al. [44] performed studies by pyrolysis-gas chromatography of thermal decomposition of 1-alkyl-3-methylimidazolium halide, revealing that nucleophilic attack by bromide anions leads to the formation of bromoethane as well as ethylene and HBr related to the cleavage of the C–N bonds at the ethyl group. Decomposition within the imidazole ring was not observed [44]. 

**Figure 6:**
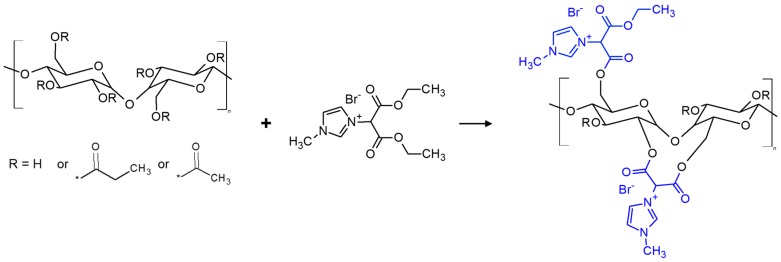
Scheme of the possible transesterification reaction between CAP and RIL.

In Figure 5(A1,A2) it can be seen that the incorporation of RIL to CAP-based materials shifts the degradation temperature to lower values. This is due to the ester bonds between RIL and CAP polymer, which are formed during polymer modification by the transesterification reaction (Figure 6). Such ester bonds possess lower stability and are more easily broken by thermal degradation [45]. Moreover, CAP-RIL membranes revealed the dependence of RIL content on the thermal stability of CAP-based membranes. In the presence of increasing content of RIL, more side chains of CAP are substituted, which is in an accordance with the systematically diminished thermal stability of CAP-RIL. 

TGA analysis revealed that the initial degradation temperature of CAP-TBC and CAP-ATBC membranes is lower in comparison with native CAP membranes and greater compared to CAP-RIL membranes (Figure 5, Table 3), whereas CAP-based membranes with 23 wt % of plasticizer start to decompose at slightly lower temperature than CAP membranes containing 9 wt % of plasticizer. For example, the onset temperatures for CAP-9-ATBC and CAP-23-ATBC membranes are 159 and 155 °C, respectively, which can be related to the initiated evaporation of plasticizers [46]. Maiza et al. reported that TGA analysis of polylactic acid (PLA) with ATBC reveals the evaporation of ATBC in the range of 284 and 335 °C due to its boiling point being equal to 173 °C. It can be seen that the type of additive has a more significant impact on the initial degradation temperature of CAP-based membranes than its content. However, it is noteworthy that tested CAP-based membranes are thermally stable up to at least 120 °C, which is an important property for polymeric membranes applied in various membrane-separation processes, including pervaporation [47]. 

**Figure 7:**
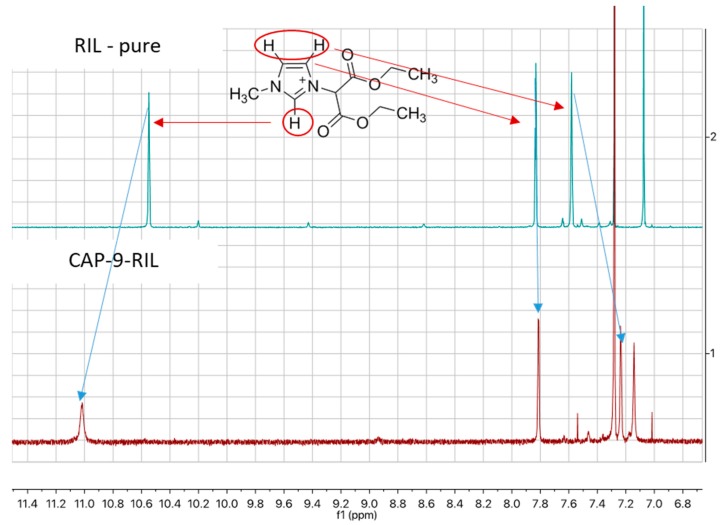
1H nuclear magnetic resonance (NMR) spectra of pure RIL and CAP-9-RIL membrane.

The occurrence of the transesterification reaction was confirmed by analyzing the 1H NMR spectra of the ionic liquid and its reaction product with CAP. The proton at C2 of the imidazole ring is de-shielded in CAP-9-RIL and shifted from 10.55 to 11.02 ppm compared to pure RIL (Figure 7). Additionally, one of the protons at C4 and C5 is shielded and shifted from 7.58 to 7.24 ppm (Figure 7). These changes in the 1H NMR spectrum indicate an occurrence of the transesterification reaction between the ionic liquid and the CAP. 

The degree of substitution of CAP ester groups in CAP-RIL blends was calculated by the following equation (Equation (9)).

(9) $$\mathrm{DS}=\frac{S1}{S2}$$

where S1 is the integral of the resonance (at 7.81 ppm) of the proton of the imidazolium ring in the ionic liquid, and S2 is the integral of the resonance (at 5.10 ppm) of the anomeric proton in a glucose unit of cellulose. 

**Figure 8:**
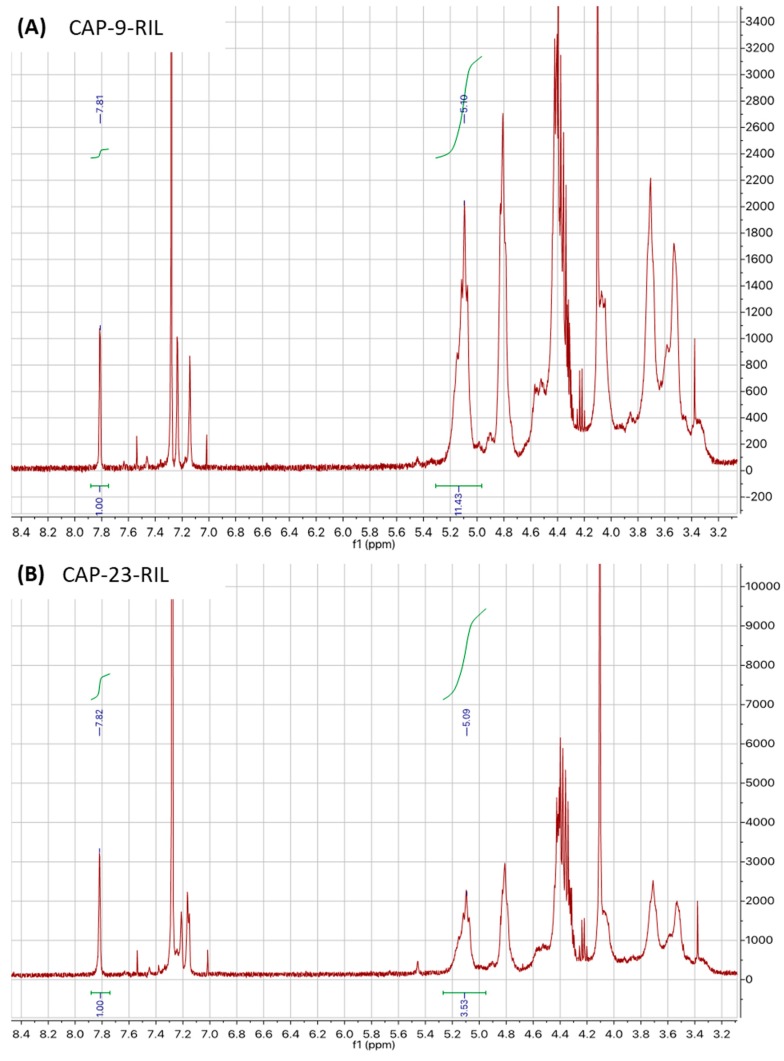
1H NMR spectra of (**A**) CAP-9-RIL; and (**B**) CAP-23-RIL membranes.

The calculated DS values are as follows, DS = 0.09 for CAP-9-RIL (Figure 8A) and DS = 0.28 for CAP-23-RIL (Figure 8B).

The maximum theoretical degree of substitution (DS_max_) was calculated as a ratio of RIL and AGU (anhydroglucose unit) molar masses taking into account the weight of RIL and CAP used to prepare CAP-RIL membranes, according to the following equation (Equation (10)):

(10) $${\mathrm{DS}}_{\max}=\frac{M_{\mathrm{RIL}}m_{\mathrm{RIL}}}{M_{\mathrm{AGU}}m_{\mathrm{CAP}}}$$

where M_AGU_ is the average molar mass of the anhydroglucose unit in CAP (325.67 g·mol^−1^); M_RIL_ is the molar mass of the ionic liquid (321.17 g·mol^−1^); and m_CAP_ and m_RIL_ are the weight (g) of CAP and RIL, respectively, in a given CAP-RIL membrane. M_AGU_ was calculated based on CAP data, provided by the producer (Eastman, USA); concerning the acetyl, propionyl, and free –OH groups content was equal to 1.3, 48.0, and 1.7 wt %, respectively.

The calculated DS_max_ value for the CAP-9-RIL membrane was equal to 0.099, which reflects the fraction of ester groups in CAP that were substituted during the transesterification reaction in the CAP-RIL membrane containing 9 wt % of RIL. On the other hand, based on the DS and DS_max_ values for the CAP-9-RIL membrane, it can be stated that 90.9% of RIL used was attached to the CAP matrix as a result of the substitution reaction. In the case of the CAP-23-RIL membrane, DS_max_ was equal to 0.295, indicating the fraction of ester groups in the CAP substituted in the CAP-23-RIL membrane. Appropriately, 95.0% of the RIL was grafted to the CAP-matrix, confirming the high efficiency of the transesterification reaction between the RIL and CAP. 

### 3.3. Equilibrium Properties

**Figure 9:**
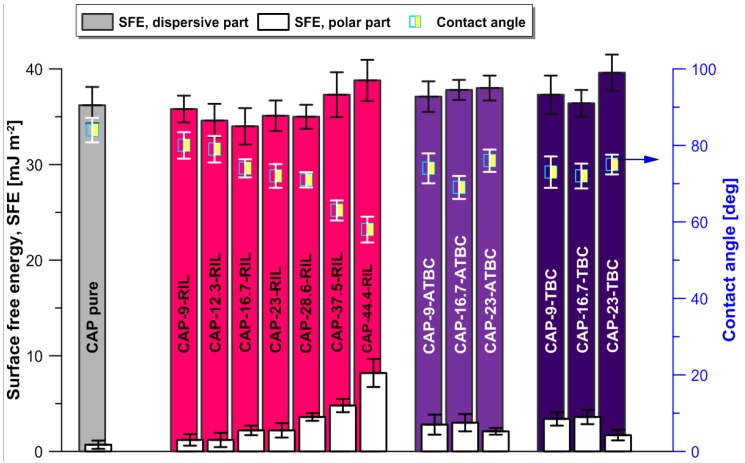
Physicochemistry of the membranes, surface free energy with its polar and dispersive components and contact angle.

Measurement of the water contact angle of the pure CAP membrane confirmed its hydrophilic character (Figure 9). The modification of CAP with an increasing content of RIL caused the decrease of water contact angle, i.e., enhancement of the hydrophilic character of the CAP-based membrane’s surface due to the hydrophilic nature of RIL. The immobilization of 44.4 wt % of RIL causes a decline in the water contact angle from 86° (for pristine CAP membrane) to 59°, which clearly demonstrates that the hydrophilicity of the CAP membrane’s surface can be effectively tuned. The presence of RIL in the CAP membranes causes an increase of the number of hydrophilic groups in the CAP, which enhances hydrogen bonding between the CAP-RIL’s surface and water drops [48]. As a consequence, CAP-RIL membranes possess improved wettability of the surface which is confirmed by increasing values of the polar component with increasing content of RIL. Simultaneously, the presence of RIL does not influence significantly the dispersive component, which reveals that the polar part, and hence the incorporation of RIL had a predominant influence on the surface hydrophilicity of the membranes tested [49] (Figure 9). Such a feasibility of changing properties of CAP-based membranes is highly desirable for the fabrication of membrane materials for hydrophilic pervaporation. 

CAP membranes plasticized with TBC and ATBC possess a hydrophilic character of the membrane’s surface; however, the type of plasticizer reveals a minor influence on the membrane surface hydrophilicity. The addition of 9 and 16.7 wt % of TBA or ATBC causes an increase in the polar part in respect to the native CAP; however, further addition of plasticizer (up to 23 wt %) results in the decrease of the polar part. 

**Figure 10:**
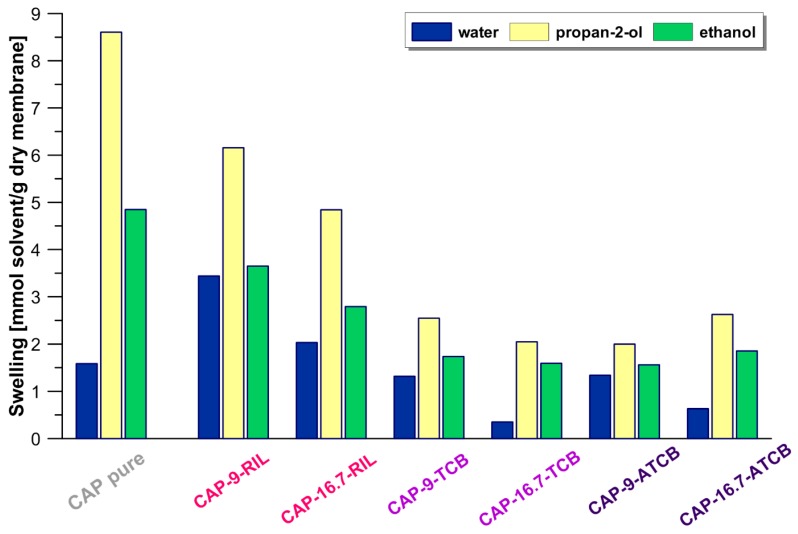
The swelling at equilibrium of pure CAP membranes and membranes doped by RIL, TBC and ATBC in contact with water, ethanol, and propan-2-ol.

The swelling ability of pure CAP membrane as well as CAP membranes modified with RIL and plasticizers (TBC or ATBC) in water, ethanol, and propan-2-ol, referred to as the molar swelling degree SD_M_ value, is presented in Figure 10. It can be seen that modification of CAP with RIL has an important impact on the CAP-RIL membrane’s swelling. The incorporation of RIL to the CAP membrane increases the swelling ability in water of CAP-based membranes with respect to the pure CAP membrane. On the other hand, the swelling of CAP-based membranes in contact with organic solvents (ethanol and propan-2-ol) decreases. Such behavior is related to the hydrophilic character of tested RIL containing bromide anions [50], which is also consistent with measurements of the water contact angle and the calculated values of SFE (Figure 9). Yun-Sheng Ye et al. [50] pointed out that the hydrophilic/hydrophobic character of an ionic liquid, and thus its affinity to solvent of different polarity, depends on the type of ionic liquid anion [50]. Due to the hydrophilic nature of the RIL1_Br studied and the low polarity of ethanol and propan-2-ol reflected by the values of relative permittivity: 25.16 and 20.18, respectively (Table 1), organic solvents are less compatible with membrane material in comparison to water (the value of relative permittivity for water is equal to 80.20—Table 1). Moreover, the water molecule reveals a better affinity and facilitated penetration of transport channels that can be formed in ionic liquid due to its smaller molar volume [51]. The presence of TBC and ATBC plasticizers did not influence the swelling behavior of CAP membranes, despite the improved hydrophilic properties of the membrane surface compared to native CAP membrane. Therefore, only CAP-RIL membranes were studied subsequently in the pervaporative separation of water-propan-2-ol feed mixture.

### 3.4. Transport and Separation Properties in Pervaporation

**Figure 11:**
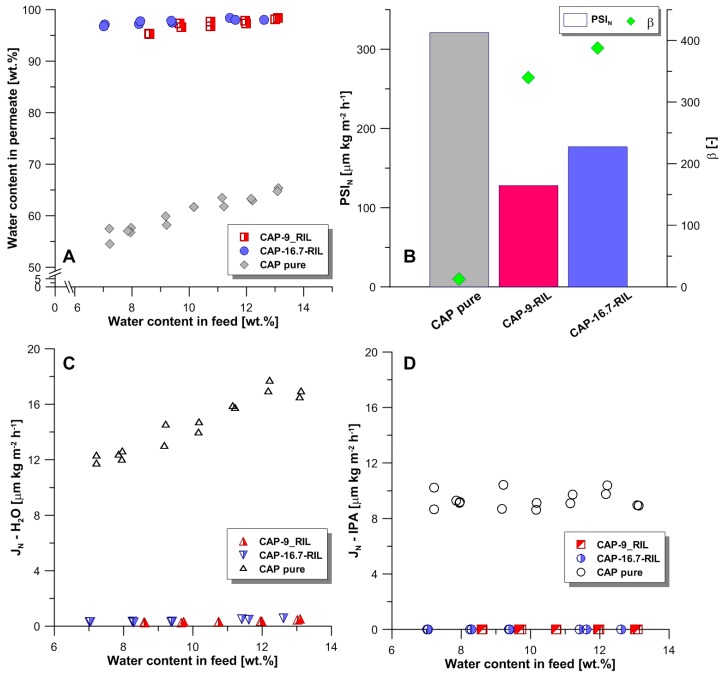
(**A**) McCabe–Thiele diagram for pristine and doped membranes; (**B**) efficiency of CAP-based membranes in contact with water-propan-2-o mixture containing 12 wt % of water (azeotropic mixture); (**C**) thickness-normalized water permeate flux; (**D**) thickness-normalized propan-2-ol permeate flux.

The effectiveness of CAP-RIL membranes in the dehydration of propan-2-ol was tested in the concentration range of water in the feed mixture between 7 and 14 wt %, and was evaluated in terms of the separation factor β—Equation (7) and the thickness-normalized process separation index (PSI_N_)—Equation (8). Special attention was paid to the separation properties of CAP-based membranes in contact with the azeotropic composition of the water-propan-2-ol feed mixture (i.e., 12 wt % of water) [52]. The results obtained revealed that pristine CAP membrane and CAP-RIL membranes are selective towards water. Increasing water quantity in the feed mixture increases the water concentration in the permeate, as presented in the McCabe–Thiele diagram (Figure 11A). The incorporation of RIL leads to a great improvement of separation factor β from 12 (pristine CAP membrane) to ca. 380 (CAP-16.7-RIL membrane) (Figure 11B). Unfortunately, the presence of RIL diminished the transport of water molecules through tested CAP-RIL membranes in comparison to the pure CAP membrane. A significant decline in the water thickness-normalized flux was observed, although the CAP-RIL membranes have improved swelling in water in contrast to pristine CAP membrane. This can be attributed to the fact that the presence of RIL diminishes the free volume of CAP-RIL membranes, which hinders the transport of permeate. As a result, a decrease in permeate fluxes and PSI_N_ for CAP-RIL membranes can be observed. However, it is noteworthy that CAP-RIL membranes are efficient in allowing propan-2-ol dehydration to break the water-propan-2-ol azeotrope. The content of water in permeate for CAP-16.7-RIL membrane was equal to 98.1 wt %. Ong and Tan [53] reported the effective separation of a ternary azeotropic mixture of ethyl acetate, ethanol, and water in contact with for buckypaper membranes immobilized with 1-butyl-3-methylimidazolium tetrafluoroborate ionic liquid and poly(vinyl alcohol) blend. The pervaporation performance at 30 °C revealed a total permeation flux of 385 g·m^−2^·h^−1^ and a separation factor equal to 247 [53]. 

## 4. Conclusions

In this work, the possibility of improving the mechanical properties of CAP-based membranes was investigated by an immobilization of RIL achieved by chemical modification of CAP with RIL. The results obtained confirmed the plasticization effect of RIL on CAP membranes and improved mechanical properties in comparison to CAP-TBC and CAP-ATBC membranes possessing commercial plasticizers. The immobilization of RIL in CAP membranes also demonstrated a predominant influence on the enhancement of the hydrophilic properties of CAP-based membranes’ surface. CAP-RIL membranes were selective in the pervaporation of water-propan-2-ol mixture in the entire concentration range of water in the feed mixture (7–14 wt %). It was found that the immobilization of RIL enhances the separation properties of CAP-based membranes, simultaneously diminishing their transport properties in the pervaporative dehydration of propan-2-ol.

## Figures and Tables

**Table 1:** Chosen physicochemical properties of water, ethanol, and propan-2-ol.

Solvent	Molar Volume (at 293.15 K)	Molar Mass	Boiling Temperature	Relative Permittivity (at 293.15 K)	Density (at 293.15 K)
	V_m_	M	T	ε	d
	[cm^3^·mol^−1^]	[g·mol^−1^]	[°C]	[-]	[g·cm^−3^]
Water	18.1	18.0	100	80.20 [25]	0.9982
Ethanol	56.9	46.1	78	25.16 [25]	0.81 *
Propan-2-ol	77.1	60.1	82	20.18 *	0.78 *

* Information supplied by the producer.

**Table 2:** Comparison of mechanical performance of CAP-RIL as well as plasticized CAP-TBC and CAP-ATBC membranes with published results for other cellulose ester-based membranes.

Type of Plasticizer	Elongation at Break (ε_max_)	Stress at Break (σ_max_)	Young’s Modulus (YM)	References
	[%]	[MPa]	[MPa]	
CAP pure	2 ± 1	50 ± 3	1710 ± 64	This work
CAP-9-RIL	65 ± 5	39 ± 01	998 ± 56	This work
CAP-9-ATBC	8 ± 6	34 ± 11	1329 ± 226	This work
CAP-9-TBC	9 ± 6	28 ± 6	1162 ± 44	This work
CAP-23-RIL	80 ± 5	28 ± 2	685 ± 62	This work
CAP-23-ATBC	61 ± 4	32 ± 2	721 ± 22	This work
CAP-23-TBC	50 ± 8	20 ± 3	571 ± 20	This work
CAB-30-TBC	30.2 ± 6.9	21.8 ± 2.3 *	-	[15]
CAB/TEOS-30-TBC	40.9 ± 13.6	25.3 ± 2.8 *	-	[15]
CAB-30-DOP	34.3 ± 2.0	28.3 ± 1.8 *	-	[15]
CAB/TEOS-30-DOP	52.1 ± 1.5	31.1 ± 1.2 *	-	[15]
CAP pure	1 ± 0	34 ± 2 *	2624 ± 169	[39]
CAP-10-Lemongrass oil	2 ± 1	25 ± 6 *	1632 ± 54	[39]
CAP-20-Lemongrass oil	2 ± 1	25 ± 8 *	1973 ± 246	[39]
CAP-10-Basil oil	1 ± 0	15 ± 4 *	1640 ± 52	[39]
CAP-20-Basil oil	-	35 ± 0 *	1603 ± 35	[39]
CAP pure	0.65	ca. 70	-	[40]
CAP-10-TCP	0.6	ca. 48	-	[40]
CAP pure	11	60 *	-	[41]
CAP-12-PTG	9	55 *	-	[41]
CAP-25-PTG	20	42 *	-	[41]
CAP-8.7-PTS	9	58 *	-	[41]
CAP-27-PTS	29	41 *	-	[41]
CAP-12-DOA	27	33 *	-	[41]

* Tensile strength.

**Table 3:** Onset temperature of the initial decomposition for CAP-RIL, CAP-TBC, and CAP-ATBC membranes.

Membrane Name	*T*_onset_ [°C]
CAP pure	351.5 ± 1.5
CAP-9-RIL	123.2 ± 1.5
CAP-12.3-RIL	120.0 ± 1.5
CAP-16.7-RIL	124.5 ± 1.5
CAP-23.0-RIL	123.8 ± 1.5
CAP-28.6-RIL	126.9± 1.5
CAP-37.5-RIL	122.9 ± 1.5
CAP-44.4-RIL	121.7 ± 1.5
CAP-9-ATBC	158.8 ± 1.5
CAP-23-ATBC	154.8 ± 1.5
CAP-9-TBC	161.4 ± 1.5
CAP-23-TBC	153.4 ± 1.5
